# Genomic surveillance of SARS-CoV-2 in Nepal

**DOI:** 10.1128/mra.00789-24

**Published:** 2024-11-12

**Authors:** Binod Khadka, Rajindra Napit, Kathie A. Mihindukulasuriya, Smita Shrestha, Ramanuj Rauniyar, Eans T. Tuladhar, Lindsay Droit, Anne M. Paredes, Runa Jha, Roji Raut, Bimalesh K. Jha, Scott A. Handley, Annie Elong-Ngono, Sujan Shresta, David Wang, Krishna D. Manandhar

**Affiliations:** 1 Central Department of Biotechnology, Tribhuvan University, Kirtipur, Kathmandu, Nepal; 2 Nirvana Biotech, Lalitpur, Nepal; 3 Department of Molecular Microbiology, Washington University School of Medicine, St. Louis, Missouri, USA; 4 Department of Pathology & Immunology, Washington University School of Medicine, St. Louis, Missouri, USA; 5 The Edison Family Center for Genome Sciences & Systems Biology, Washington University School of Medicine, St. Louis, Missouri, USA; 6 Maharajgunj Medical Campus, Tribhuvan University, Kathmandu, Nepal; 7 National Public Health Laboratory, Teku, Kathmandu, Nepal; 8 Center for Vaccine Innovation, La Jolla Institute for Immunology, La Jolla, California, USA; Queens College Department of Biology, Queens, New York, USA

**Keywords:** COVID-19, whole-genome sequencing, coronavirus, Illumina

## Abstract

During the COVID-19 pandemic, Nepal, like other countries, faced emerging SARS-CoV-2 variants. To evaluate the circulating variants, 278 samples collected between September 2021 and March 2022 were sequenced in the country. From these, 229 high-quality genomes were obtained (82.97% Omicron and 17.03% Delta) highlighting the genomic diversity of SARS-CoV-2 in Nepal.

## ANNOUNCEMENT

During the COVID-19 pandemic caused by SARS-CoV-2, a positive-sense, single-stranded RNA virus of the family *Coronaviridae* and genus *Betacoronavirus* (1), the availability of complete, high-quality viral genome sequence data was limited in Nepal (2, 3).

A total of 278 nasopharyngeal swab samples from the government approved National Public Health laboratory (collected from different parts of the country) and KM-TU Biotech Covid Lab (collected from Kathmandu) from September 2021 to March 2022 were studied. The samples were stored at −80°C until use. RNA was extracted using Chemagic Prime Viral RNA kit (CMG-1449) on a Perkin Elmer Chemagic 360 instrument. Real Time Multiplex RT-PCR was performed using the Liferiver Novel Coronavirus (2019-nCoV) Real Time Multiplex RT-PCR Kit (Ref no.: RR-0479-02), and SARS-CoV-2-positive samples with *C*_*t*_ values less than 30 were used for sequencing.

Following Illumina COVIDSeq protocol (#1000000126053-04), the SARS-CoV-2 genome of each sample was amplified using CPP1 and CPP2 ARTIC v3 pools of primers (https://github.com/artic-network/artic-ncov2019/blob/master/primer_schemes/nCoV-2019/V3/nCoV-2019.tsv). Amplicons from each of the two PCR reactions per sample were pooled and indexed with “IDT for Illumina-PCR Indexes” (unique index per sample) using on-bead tagmentation. Each indexed sample was then pooled for the final library, followed by quality check with Agilent Bioanalyzer (Kit Cat. 50671504) and quantified with Qubit 3.0 (Kit Cat. Q32851). The final library was diluted to 4 nM and denatured. The sample was loaded on the sequencer at 10 pmol. Sequencing was done on Illumina MiSeq with the 2 × 300 bp paired-end protocol using MiSeqV3 reagent kit (MS-102–3003) at the IVDRL, Tribhuvan University, Nepal.

The 229 high-quality genomic sequences (Table 1) were analyzed using the CDC SARS-CoV-2 BFX-UT_ARTIC_Illumina pipeline (https://github.com/CDCgov/SARS-CoV-2_Sequencing/tree/master/protocols/BFX-UT_ARTIC_Illumina). The Illumina reads were mapped (bwa-mem version 0.7.17-r1188) to the SARS-CoV-2 reference genome (MN908947.3), sorted, and unmapped reads were removed using samtools (version 1.17 using htslib 1.18). The total number of reads and the average sequencing depth (201–4,766) per genome are listed in Table 1. Primers were trimmed, and the consensus genome (39 complete and 190 nearly complete coding sequence) was generated using iVar version 1.4.2 (4). Variants were called with iVar version 1.4.2 followed by LoFreq version 2.1.5 (5), then annotated with snpEff version 5.1f and snpSift version 4.3t (6). The sequences were concatenated, and lineages were assigned using Pangolin version 1.1.14 (7) and NextClade version 2.14.0 (8), combined in R version 4.2.2. Sequences with more than 3,000 Ns compared to the reference genome were excluded. Full genomes were aligned using MAFFT version 0.7 (9), and a phylogenetic tree was generated with Augur version 17.7.0 from Nextstrain (10). The tree was visualized and edited in iTOL version 5 (11), with clade labels added in Microsoft Preview. Default settings were used for all these software unless otherwise specified.

**TABLE 1:** Details of SARS-CoV-2 samples, sequence analysis, and hyperlinks of the study

S. No.	Sample name	Collection_Date	Hyperlinked SRA accession ID	GenBank accession ID	GC content (%)	Lineage (Nextclade)	clade_who	Length	qc.overallScore (Nextclade)	Genome coverage (%) (Nextclade)	Number of reads	Sequencing depth
1	BB466	2021-12	SRR29872869	PP825474	39	BA.1.17.2	Omicron	29,760	60.61	91.6	139,487	605.13
2	qc5158	2022-01	SRR29872868	PP825475	39.80	BA.2	Omicron	29,805	11.56	95.7	236,261	1,470.6
3	qc8160	2022-02	SRR29872857	PP825476	39.40	BA.2	Omicron	29,648	18.46	94.4	141,771	725.48
4	qc8213	2022-02	SRR29872846	PP825477	39	BA.2.10	Omicron	29,636	14.08	94.8	278,256	1,550.1
5	qc8215	2022-02	SRR29872835	PP825478	38.70	BA.2	Omicron	29,797	36.76	93.3	255,107	1,525
6	qc8219	2022-02	SRR29872824	PP825479	39.40	BA.2.10	Omicron	29,621	19.43	94.2	245,897	1,388.8
7	qc8220	2022-02	SRR29872823	PP825480	38.50	BA.2.10	Omicron	29,781	5.58	96.6	182,429	1,242.1
8	qc8221	2022-02	SRR29872822	PP825481	39	BA.2	Omicron	29,640	4.92	96.2	256,586	1,565.4
9	qc8228	2022-02	SRR29872821	PP825482	41.30	BA.2	Omicron	29,755	64.59	91.3	176,481	905.09
10	qc8249	2022-01	SRR29872820	PP825483	38.80	BA.2	Omicron	29,708	94.81	89.6	169,426	925.52
11	qc8258	2022-02	SRR29872867	PP825484	38.80	BA.2	Omicron	29,778	13.55	95.3	190,765	1,127.2
12	qc8260	2022-02	SRR29872866	PP825485	37.90	BA.2.10	Omicron	29,602	54.21	91.4	225,175	1,652.3
13	qc8261	2022-02	SRR29872865	PP825486	38.20	BA.2.10	Omicron	29,703	15.12	94.9	284,906	1,597.7
14	qc8273	2022-01	SRR29872864	PP825487	38.80	BA.2	Omicron	29,678	16.84	94.6	177,154	1,098.1
15	qc8274	2022-01	SRR29872863	PP825488	39.10	BA.2	Omicron	29,747	44.49	92.5	134,298	751.64
16	qc8280	2022-01	SRR29872862	PP825489	37.90	BA.2.10	Omicron	29,638	16.72	94.5	281,714	1,847.9
17	qc8281	2022-01	SRR29872861	PP825490	39.40	BA.2.2	Omicron	29,805	12.99	95.5	321,001	1,749.7
18	qc8317	2022-02	SRR29872860	PP825491	38.80	BA.2.10	Omicron	29,637	17.36	94.4	130,560	714.6
19	qc8319	2022-02	SRR29872859	PP825492	38.40	BA.2	Omicron	29,774	17.48	95	175,423	1,061.8
20	qc8321	2022-02	SRR29872858	PP825493	37.90	BA.2	Omicron	29,799	60.72	91.7	238,459	1,453.1
21	qc8322	2022-02	SRR29872856	PP825494	39	BA.2.2	Omicron	29,631	17.53	94.6	162,709	1,035.8
22	qc8323	2022-02	SRR29872855	PP825495	39.30	BA.2	Omicron	29,697	18.02	94.6	169,050	951.75
23	qc8324	2022-02	SRR29872854	PP825496	41.80	BA.2	Omicron	29,647	9.86	95.4	347,504	1,853.2
24	qc8326	2022-02	SRR29872853	PP825497	41.10	BA.2	Omicron	29,642	8.09	95.6	398,228	2,255.1
25	qc8327	2022-02	SRR29872852	PP825498	39	BA.2	Omicron	29,660	71.68	90.6	247,540	1,428.3
26	qc8338	2022-02	SRR29872851	PP825499	39.80	BA.2.10	Omicron	29,713	61.01	91.4	239,720	1,205.3
27	qc8342	2022-02	SRR29872850	PP825500	40.90	BA.2	Omicron	29,578	49.26	91.7	236,197	1,134.4
28	qc8344	2022-02	SRR29872849	PP825501	38.60	BA.2	Omicron	29,634	8.13	95.6	271,237	1,608.3
29	QC8512	2022-03	SRR29872848	PP825502	38.70	BA.2	Omicron	29,729	0.69	99.6	154,684	1,217.7
30	QC8514	2022-03	SRR29872847	PP825503	41.30	BA.2	Omicron	29,722	30.41	93.6	77,197	456.37
31	QC8516	2022-03	SRR29872845	PP825504	39.30	BA.2.10	Omicron	29,729	0	98.7	152,397	1,006
32	QC8532	2022-03	SRR29872844	PP825505	42.20	BA.2	Omicron	29,774	14.1	95.4	170,158	999.32
33	QC8534	2022-03	SRR29872843	PP825506	39.40	BA.2.2	Omicron	29,776	0.98	97.9	108,161	718.66
34	QC8538	2022-03	SRR29872842	PP825507	39.90	BA.2	Omicron	29,729	36.22	93.2	171,852	1,127.5
35	QC8598	2022-03	SRR29872841	PP825508	38.40	BA.2	Omicron	29,775	0	99.4	140,043	1,101.9
36	QC8629	2022-03	SRR29872840	PP825509	39.90	BA.2	Omicron	29,781	1.56	99.7	139,410	956.51
37	QC8630	2022-03	SRR29872839	PP825510	44.30	BA.2.10	Omicron	29,441	80.47	89.5	88,085	381.77
38	QC8636	2022-03	SRR29872838	PP825511	38.80	BA.2	Omicron	29,779	0	99	124,409	956.03
39	QC8638	2022-03	SRR29872837	PP825512	43.80	BA.2	Omicron	29,635	70	90.7	83,769	398.32
40	QC8641	2022-03	SRR29872836	PP825513	40.20	BA.2.10	Omicron	29,698	9.96	95.6	73,226	497.95
41	QC8644	2022-03	SRR29872834	PP825514	43.20	BA.2	Omicron	29,682	17.39	94.7	117,215	591.27
42	QC8651	2022-03	SRR29872833	PP825515	39.80	BA.2	Omicron	29,771	38.81	93.1	131,622	945.49
43	QC8661	2022-03	SRR29872832	PP825516	39.70	BA.2	Omicron	29,776	5.02	98	83,146	577.5
44	QC8663	2022-03	SRR29872831	PP825517	43.90	BA.2	Omicron	29,741	39.46	92.9	70,942	291.18
45	QC8664	2022-03	SRR29872830	PP825518	39.40	BA.2	Omicron	29,723	2.48	97.4	145,775	1,031.2
46	QC8665	2022-03	SRR29872829	PP825519	40.20	BA.2	Omicron	29,729	1.02	97.7	137,181	929.15
47	QC8667	2022-03	SRR29872828	PP825520	44.40	BA.2.10.1	Omicron	29,755	31.57	93.5	112,736	433.42
48	QC8668	2022-03	SRR29872827	PP825521	40.50	BA.2.10	Omicron	29,739	0	99.5	100,208	657.97
49	QC8710	2022-03	SRR29872826	PP825522	41	BA.2	Omicron	29,779	51.57	92.3	106,539	740.48
50	QC8711	2022-03	SRR29872825	PP825523	40.20	BA.2	Omicron	29,781	1.37	97.7	137,732	998.91
51	QC8712	2022-03	SRR29874965	PP825524	39.90	BA.2	Omicron	29,709	55.25	91.8	183,189	1,347.3
52	QC8714	2022-03	SRR29874964	PP825525	44	BA.2	Omicron	29,568	58.27	91.2	88,203	345.46
53	QC8731	2022-03	SRR29874934	PP825526	38.60	BA.2.10	Omicron	29,789	0	99.8	180,103	1,353.3
54	QC8732	2022-03	SRR29874923	PP825527	39.10	BA.2	Omicron	29,773	0	99.2	134,732	998.56
55	QC8733	2022-03	SRR29874959	PP825528	39.70	BA.2	Omicron	29,744	0.02	98.5	62,387	437.29
56	QC8734	2022-03	SRR29874948	PP825529	43.40	BA.2	Omicron	29,779	1.6	97.6	63,998	361.41
57	QC8735	2022-03	SRR29874947	PP825530	50.20	BA.2	Omicron	29,755	77.51	90.6	61,243	276.08
58	QC8736	2022-03	SRR29874946	PP825531	40.50	BA.2	Omicron	29,779	0	99.7	110,527	745.68
59	QC8737	2022-03	SRR29874945	PP825532	38.40	BA.2.3	Omicron	29,729	0	99.2	106,569	831.41
60	QC8738	2022-03	SRR29874943	PP825533	38.20	BA.2	Omicron	29,776	0.09	98.5	97,165	770.57
61	QC8768	2022-03	SRR29874963	PP825534	38.80	BA.2	Omicron	29,773	13.36	95.4	83,925	639.71
62	QC8803	2022-02	SRR29874944	PP825535	38.20	BA.2	Omicron	29,729	0	98.8	150,622	1,178.4
63	QC8812	2022-02	SRR29874942	PP825536	39.10	BA.2	Omicron	29,726	22.54	94.3	103,461	742.99
64	QC8837	2022-02	SRR29874941	PP825537	38.50	BA.2	Omicron	29,745	0	98.8	106,001	808.3
65	QC8844	2022-03	SRR29874940	PP825538	38.70	BA.2	Omicron	29,769	5.57	96.6	60,147	455.91
66	QC8845	2022-03	SRR29874939	PP825539	41.50	BA.2	Omicron	29,729	11.64	95.5	76,449	440.91
67	QC8847	2022-03	SRR29874938	PP825540	40	BA.2.10	Omicron	29,790	7.23	96.4	79,020	542.74
68	QC8849	2022-03	SRR29874937	PP825541	40.10	BA.2	Omicron	29,729	8.11	96	122,295	813.73
69	QC8850	2022-03	SRR29874936	PP825542	38.20	BA.2	Omicron	29,766	2.75	97.2	97,401	769.71
70	QC8851	2022-03	SRR29874935	PP825543	38.40	BA.2.10	Omicron	29,792	3.72	97.1	80,651	648.05
71	QC8852	2022-03	SRR29874933	PP825544	39.40	BA.2	Omicron	29,777	2.81	97.2	163,295	1,178.1
72	QC8853	2022-03	SRR29874932	PP825545	38.10	BA.2	Omicron	29,805	85.87	90.4	83,370	630.61
73	QC8854	2022-03	SRR29874931	PP825546	38.50	BA.2	Omicron	29,781	40.68	93	112,793	856.61
74	QC8856	2022-03	SRR29874930	PP825547	38.40	BA.2	Omicron	29,778	93.59	90	66,083	500.9
75	QC8858	2022-03	SRR29874929	PP825548	38.90	BA.2	Omicron	29,781	91.52	90.1	97,493	710.69
76	QC8868	2022-03	SRR29874928	PP825549	41.20	BA.2	Omicron	29,779	7.8	96.2	147,903	909.59
77	QC8870	2022-03	SRR29874927	PP825550	38.40	BA.2	Omicron	29,781	13.31	95.5	106,570	770.01
78	QC8911	2022-03	SRR29874926	PP825551	39.40	BA.2	Omicron	29,781	8.28	96.2	127,597	948.84
79	QC8912	2022-03	SRR29874925	PP825552	42.60	BA.2	Omicron	29,744	94.02	89.9	63,355	366.15
80	QC8913	2022-03	SRR29874924	PP825553	40.70	BA.2	Omicron	29,729	80.67	90.5	82,204	584.12
81	QC8914	2022-03	SRR29874922	PP825554	40.50	BA.2	Omicron	29,781	3.78	97	89,511	637.07
82	QC8916	2022-03	SRR29874921	PP825555	39.30	BA.2	Omicron	29,805	42.35	92.9	106,977	761.39
83	QC8917	2022-03	SRR29874920	PP825556	42.50	BA.2	Omicron	29,770	69.31	91.1	114,848	615.2
84	QC8920	2022-03	SRR29874919	PP825557	41.30	BA.2	Omicron	29,773	71.31	91.1	112,709	765.84
85	QC8927	2022-03	SRR29874918	PP825558	38.20	BA.2	Omicron	29,755	78.81	90.6	95,313	751.59
86	QC8938	2022-03	SRR29874917	PP825559	41	BA.2	Omicron	29,768	30.54	93.7	103,909	672.93
87	QC8944	2022-03	SRR29874916	PP825560	39.80	BA.2.31.1	Omicron	29,771	30.54	93.7	131,260	973.09
88	QC8946	2022-03	SRR29874962	PP825561	41	BA.2	Omicron	29,779	7.7	96.3	150,787	1,019
89	QC8948	2022-03	SRR29874961	PP825562	40.30	BA.2	Omicron	29,770	91.03	90	66,787	490.69
90	QC8957	2022-03	SRR29874960	PP825563	40.50	BA.2	Omicron	29,781	3.93	97	251,044	1,876.1
91	QC8958	2022-03	SRR29874958	PP825564	42.60	BA.2	Omicron	29,771	22.33	94.5	252,585	1,414
92	QC8965	2022-03	SRR29874957	PP825565	39.20	BA.2	Omicron	29,787	4.43	98.5	276,743	2,243.1
93	QC8966	2022-03	SRR29874956	PP825566	40.50	BA.2.50	Omicron	29,814	0.02	98.7	237,570	1,813.3
94	QC8967	2022-03	SRR29874955	PP825567	40.50	BA.2.12.1	Omicron	29,781	6.7	96.4	343,467	2,534.5
95	QC8968	2022-03	SRR29874954	PP825568	40.70	BA.2.50	Omicron	29,781	12.46	95.6	110,853	818.2
96	QC8969	2022-03	SRR29874953	PP825569	40.40	BA.2	Omicron	29,781	0	98.9	175,990	1,358
97	QC8975	2022-03	SRR29874952	PP825570	41.20	BA.2	Omicron	29,779	19.43	94.8	124,140	897.66
98	QC8979	2022-03	SRR29874951	PP825571	38.90	BA.2	Omicron	29,760	82.54	90.4	110,570	895.92
99	QC8981	2022-03	SRR29874950	PP825572	39.60	BA.2.3	Omicron	29,744	19.62	94.6	213,190	1,735.1
100	QC9031	2022-03	SRR29874949	PP825573	38.40	BA.2.3	Omicron	29,776	1.49	97.6	120,816	1,087.3
101	QC9032	2022-03	SRR29875192	PP825574	38.30	BA.2.3	Omicron	29,763	10.7	95.7	139,954	1,237.8
102	QC9033	2022-03	SRR29875191	PP825575	38.60	BA.2.3	Omicron	29,781	0	98.8	139,818	1,228.1
103	QC9034	2022-03	SRR29875180	PP825576	38.30	BA.2.9	Omicron	29,799	36.58	93.3	164,730	1,474.4
104	QC9035	2022-03	SRR29875169	PP825577	39.20	BA.2	Omicron	29,779	9.66	96	174,932	1,504.9
105	QC9036	2022-03	SRR29875158	PP825578	40.50	BA.2	Omicron	29,781	0.01	98.7	190,449	1,516.8
106	QC9037	2022-03	SRR29875147	PP825579	42.10	BA.2	Omicron	29,779	12.22	95.6	258,998	1,845.1
107	QC9038	2022-03	SRR29875146	PP825580	39.90	BA.2	Omicron	29,781	2.74	97.3	237,240	1,990.9
108	QC9039	2022-03	SRR29875145	PP825581	40.80	BA.2	Omicron	29,775	0.1	98.5	154,325	1,211.5
109	QC9051	2022-03	SRR29875144	PP825582	38.20	BA.2	Omicron	29,734	0.17	99.6	183,635	1,857.9
110	QC9052	2022-03	SRR29875143	PP825583	38.40	BA.2	Omicron	29,734	0.54	97.9	256,284	2,459.7
111	QC9053	2022-03	SRR29875190	PP825584	40.20	BA.2	Omicron	29,729	0	99.1	218,645	1,753.8
112	QC9055	2022-03	SRR29875189	PP825585	42.60	BA.2	Omicron	29,729	0.42	98	190,770	1,236.4
113	QC9056	2022-03	SRR29875188	PP825586	40.20	BA.2	Omicron	29,786	17.36	99.6	271,489	2,311
114	QC9057	2022-03	SRR29875187	PP825587	39.30	BA.2	Omicron	29,729	0.68	97.8	187,485	1,645.5
115	QC9058	2022-03	SRR29875186	PP825588	41.80	BA.2	Omicron	29,729	19.16	94.6	204,331	1,099.9
116	QC9059	2022-03	SRR29875185	PP825589	40.90	BA.4.2	Omicron	29,714	22.06	94.4	101,473	604.09
117	QC9081	2022-03	SRR29875184	PP825590	38.20	BA.2	Omicron	29,729	0.04	98.4	159,177	1,571.5
118	QC9097	2022-03	SRR29875183	PP825591	38.50	BA.2	Omicron	29,675	9.77	95.6	229,436	1,808.7
119	QC9116	2022-03	SRR29875182	PP825592	39.30	BA.2	Omicron	29,809	0.62	98.1	234,880	2,084.3
120	QC9117	2022-03	SRR29875181	PP825593	40.70	BA.2.10	Omicron	29,781	13.23	95.5	132,163	1,049
121	SQ1513	2021-11	SRR29875179	PP825594	38.90	B.1.617.2	Delta	29,821	68.38	91.4	177,005	789.19
122	SQ1529	2021-12	SRR29875178	PP825595	39	B.1.617.2	Delta	29,855	46.04	94.4	518,055	2,193.2
123	SQ1537	2021-12	SRR29875177	PP825596	38.60	B.1.617.2	Delta	29,806	16.6	95.2	198,401	891.36
124	sq1539	2021-12	SRR29875176	PP825597	38.40	B.1.617.2	Delta	29,454	51.05	91.1	140,714	597.45
125	SQ1580	2021-12	SRR29875175	PP825598	38.60	B.1.617.2	Delta	29,821	60.8	91.8	455,614	2,032.7
126	SQ1635	2021-12	SRR29875174	PP825599	39	B.1.617.2	Delta	29,614	56.11	91.4	113,343	498.8
127	sq1637	2021-12	SRR29875173	PP825600	38.40	B.1.617.2	Delta	29,819	1.72	97.6	171,968	766.33
128	sq1640	2021-12	SRR29875172	PP825601	38.40	B.1.617.2	Delta	29,860	58.85	92	313,925	1,327.5
129	SQ1651	2021-12	SRR29875171	PP825602	39.50	B.1.617.2	Delta	29,785	40.97	93.2	179,134	769.07
130	sq1654	2021-12	SRR29875170	PP825603	38.70	B.1.617.2	Delta	29,814	16.75	96.2	145,600	1,070.6
131	sq1655	2021-12	SRR29875168	PP825604	39.40	B.1.617.2	Delta	29,794	3.39	97.5	254,578	1,110.5
132	SQ1665	2021-12	SRR29875167	PP825605	38.50	B.1.617.2	Delta	29,819	10.78	96.5	133,604	603.22
133	SQ1667	2021-12	SRR29875166	PP825606	39.20	B.1.617.2	Delta	29,806	23.02	96	115,505	495.66
134	sq1672	2021-12	SRR29875165	PP825607	38	B.1.617.2	Delta	29,492	1.64	97.4	207,546	912.01
135	SQ1680	2021-11	SRR29875164	PP825608	39.90	B.1.617.2	Delta	29,806	42.7	93.3	139,687	585.89
136	SQ1682	2021-11	SRR29875163	PP825609	39.70	B.1.617.2	Delta	29,798	72.56	91.1	207,047	873.18
137	SQ1689	2021-11	SRR29875162	PP825610	39	B.1.617.2	Delta	29,820	21.81	94.8	206,476	900.04
138	sq1690	2021-11	SRR29875161	PP825611	38.40	AY.4	Delta	29,691	88.05	90.2	81,881	348.81
139	sq1702	2021-11	SRR29875160	PP825612	38.80	AY.75	Delta	29,817	30.92	96.6	346,627	1,501.6
140	sq1703	2021-11	SRR29875159	PP825613	38.50	B.1.617.2	Delta	29,735	81.46	90.4	81,599	348.63
141	sq1705	2021-11	SRR29875157	PP825614	38.80	B.1.617.2	Delta	29,821	22.86	94.7	185,534	810.47
142	SQ1707	2021-11	SRR29875156	PP825615	39.80	B.1.617.2	Delta	29,811	38.93	95.9	145,128	616.64
143	SQ1713	2021-11	SRR29875155	PP825616	41.20	B.1.617.2	Delta	29,785	52.98	92.2	190,191	749.53
144	SQ1720	2021-12	SRR29875154	PP825617	39.30	B.1.617.2	Delta	29,889	12.46	97.2	1,097,224	4,765.7
145	sq1723	2021-12	SRR29875153	PP825618	38.80	B.1.617.2	Delta	29,827	58.95	91.8	223,543	954.62
146	sq1729	2021-12	SRR29875152	PP825619	38.30	B.1.617.2	Delta	29,738	91.99	92.6	117,549	499.5
147	sq1742	2021-12	SRR29875151	PP825620	38.20	B.1.617.2	Delta	29,636	13.05	95.1	265,739	1,152.6
148	SQ1752	2021-12	SRR29875150	PP825621	38.90	B.1.617.2	Delta	29,878	76.47	94.9	355,941	1,565.4
149	SQ1755	2021-12	SRR29875149	PP825622	39.20	B.1.617.2	Delta	29,885	10.48	96.1	808,264	3,431.5
150	sq1780	2021-12	SRR29875148	PP825623	38.10	B.1.617.2	Delta	29,315	90.31	88.9	110,417	470.77
151	sq1819	2021-12	SRR29876922	PP825624	38.20	B.1.617.2	Delta	29,573	70.81	90.5	46,018	200.75
152	SQ1841	2021-12	SRR29876921	PP825625	38.90	B.1.617.2	Delta	29,887	21.63	95.4	960,625	4,271
153	SQ1868	2021-12	SRR29876862	PP825626	38.90	B.1.617.2	Delta	29,748	58.66	91.9	138,073	599.77
154	SQ1871	2021-12	SRR29876851	PP825627	42.60	B.1.617.2	Delta	29,871	67.18	91.5	546,357	1,719.4
155	SQ1874	2021-12	SRR29876916	PP825628	38.70	B.1.617.2	Delta	29,819	47.9	93.8	154,688	680.96
156	SQ5043	2021-09	SRR29876905	PP825629	38.50	B.1.617.2	Delta	29,819	3.82	97.4	974,178	4,394.8
157	sq5149	2022-01	SRR29876894	PP825630	41.40	BA.2	Omicron	29,584	88.01	89.5	163,335	744.84
158	sq5150	2022-01	SRR29876883	PP825631	39	BA.2	Omicron	29,755	23.47	94.2	218,801	1,082.5
159	sq5151	2022-01	SRR29876873	PP825632	39.40	BA.2.2	Omicron	29,781	5.98	96.6	173,514	1,000.1
160	sq5152	2022-01	SRR29876871	PP825633	38.90	BA.2.2	Omicron	29,802	6.61	96.4	210,124	1,004
161	sq5153	2022-01	SRR29876920	PP825634	39.10	AY.76	Delta	29,598	160.68	89.1	182,923	914.95
162	sq5154	2022-01	SRR29876872	PP825635	39.40	BA.2	Omicron	29,755	25.15	94.1	154,101	892.92
163	sq5157	2022-01	SRR29876870	PP825636	39.60	BA.2.10	Omicron	29,755	38.85	93	208,367	869.91
164	sq5207	2022-01	SRR29876869	PP825637	38.60	BA.1.1	Omicron	29,752	69.2	91.1	237,055	1,295.5
165	sq5208	2022-01	SRR29876868	PP825638	38.60	BA.1.1	Omicron	29,727	74.73	90.7	126,607	583.6
166	sq5209	2022-01	SRR29876867	PP825639	38.10	BA.1.1	Omicron	29,752	46.34	92.4	253,489	1,285.2
167	sq5215	2022-01	SRR29876866	PP825640	38.50	BA.2.10	Omicron	29,632	31.53	93.1	210,497	1,052.7
168	sq5218	2022-01	SRR29876865	PP825641	38.40	BA.2.10	Omicron	29,807	4.39	96.9	278,136	1,430.4
169	sq5220	2022-01	SRR29876864	PP825642	38.10	BA.1.1	Omicron	29,752	23.83	94.3	253,216	1,516.1
170	sq5224	2022-01	SRR29876863	PP825643	38	BA.2.10	Omicron	29,792	14.72	95.3	229,726	1,252.5
171	sq5226	2022-01	SRR29876861	PP825644	38.60	BA.2.10	Omicron	29,772	15.97	95	348,822	2,029.8
172	sq5230	2022-01	SRR29876860	PP825645	39	BA.2.10	Omicron	29,704	28.13	93.6	347,876	1,944.6
173	sq5234	2022-01	SRR29876859	PP825646	38	BA.2.10	Omicron	29,772	6.07	96.4	289,439	1,610.9
174	sq5236	2022-01	SRR29876858	PP825647	39	BA.2.10	Omicron	29,755	32.79	93.4	146,310	768.35
175	sq5240	2022-01	SRR29876857	PP825648	38.10	BA.2.10	Omicron	29,755	24.41	94.1	266,493	1,470.9
176	sq5248	2022-01	SRR29876856	PP825649	38.50	BA.2.10	Omicron	29,699	4.71	96.4	233,513	1,462.2
177	sq5254	2022-01	SRR29876855	PP825650	39	BA.2	Omicron	29,742	11.56	95.5	200,688	1,111.9
178	sq5257	2022-01	SRR29876854	PP825651	38.90	BA.2.10	Omicron	29,797	30.99	93.7	131,641	824.97
179	sq5260	2022-01	SRR29876853	PP825652	38.60	BA.2.10	Omicron	29,584	25.15	93.5	271,286	1,550.4
180	sq5303	2022-01	SRR29876852	PP825653	38.40	BA.2	Omicron	29,773	1.11	97.7	384,010	2,044.3
181	sq5304	2022-01	SRR29876850	PP825654	38.50	BA.1.1	Omicron	29,752	8.26	96	267,500	1,395.2
182	sq5373	2022-01	SRR29876849	PP825655	38.70	BA.1	Omicron	29,798	632.37	96.3	259,655	1,661
183	sq5379	2022-01	SRR29876848	PP825656	39.30	BA.2	Omicron	2,9652	28.48	93.4	281,980	1,290.9
184	sq5392	2022-01	SRR29876847	PP825657	39	BA.2	Omicron	29,781	0.15	98.4	308,318	1,936.6
185	sq5400	2022-01	SRR29876846	PP825658	38.20	BA.2	Omicron	29,805	0.68	98	220,174	1,302.4
186	sq5406	2022-01	SRR29876845	PP825659	39	BA.2	Omicron	29,805	5.32	96.7	322,155	1,746.6
187	sq5408	2022-01	SRR29876844	PP825660	38.70	BA.2	Omicron	29,803	11.53	95.7	215,785	1,091.3
188	sq5452	2022-01	SRR29876919	PP825661	38.50	BA.1.1	Omicron	29,804	0.67	98	293,342	1,820
189	sq5476	2022-01	SRR29876918	PP825662	38.30	BA.2.10	Omicron	29,664	6.74	95.9	166,667	864.34
190	sq5489	2022-01	SRR29876917	PP825663	37.90	BA.2	Omicron	29,807	2.85	97.2	243,357	1,375.1
191	sq5498	2022-01	SRR29876915	PP825664	38.60	BA.2	Omicron	29,755	34.81	93.3	308,560	1,624.5
192	sq5502	2022-01	SRR29876914	PP825665	38.90	BA.1	Omicron	29,611	32.75	93	193,930	1,104.5
193	sq5509	2022-01	SRR29876913	PP825666	37.90	B.1.617.2	Delta	29,769	13.83	97.1	239,876	1,447.8
194	sq5514	2022-01	SRR29876912	PP825667	38.40	BA.2	Omicron	29,799	3.53	97	367,112	2,013.3
195	sq5519	2022-01	SRR29876911	PP825668	38.50	BA.2.10	Omicron	29,755	81.47	90.4	249,932	1,436.6
196	sq5526	2022-01	SRR29876910	PP825669	39.60	BA.2	Omicron	29,755	32.79	93.4	216,286	990.24
197	sq6155	2022-02	SRR29876909	PP825670	38.90	BA.2	Omicron	29,626	14.64	94.7	230,171	1,574.4
198	sq6157	2022-02	SRR29876908	PP825671	39.70	BA.2.10	Omicron	29,660	15.04	94.8	266,996	1,628.6
199	sq6158	2022-02	SRR29876907	PP825672	41	BA.2.10	Omicron	29,653	15.38	94.7	171,948	939.71
200	sq6164	2022-02	SRR29876906	PP825673	41.30	BA.2.10	Omicron	29,701	9.16	95.7	228,609	1,208.5
201	sq6167	2022-02	SRR29876904	PP825674	39.20	BA.2	Omicron	29,704	12.46	95.2	232,901	1,288.5
202	sq6168	2022-02	SRR29876903	PP825675	39.30	BA.2.10	Omicron	29,806	12.17	95.6	309,913	1,877.6
203	sq6218	2022-02	SRR29876902	PP825676	38.80	BA.2	Omicron	29,628	12.04	95	307,585	1,864.9
204	sq6220	2022-02	SRR29876901	PP825677	38.30	BA.2	Omicron	29,584	13.31	94.7	175,806	1,171.7
205	sq6224	2022-02	SRR29876900	PP825678	39.10	BA.2	Omicron	29,805	11.76	95.7	345,097	2,066.6
206	sq6227	2022-02	SRR29876899	PP825679	38.80	BA.2	Omicron	29,779	19.85	94.6	257,662	1,601.1
207	SQ6292	2022-03	SRR29876898	PP825680	38.90	BA.2.12.1	Omicron	29,780	4.06	96.9	234,714	1,846
208	SQ6294	2022-04	SRR29876897	PP825681	39.50	BA.2	Omicron	29,780	13.34	95.5	265,917	1,967
209	SQ6296	2022-04	SRR29876896	PP825682	41.50	BA.2.43	Omicron	29,728	35.51	93.2	96,169	609.25
210	SQ6297	2022-04	SRR29876895	PP825683	40.50	BA.2.78	Omicron	29,779	6.38	96.5	222,689	1,663.5
211	SQ6298	2022-04	SRR29876893	PP825684	38.50	BA.2.43	Omicron	29,775	2.64	97.3	222,151	1,798.6
212	SQ6299	2022-04	SRR29876892	PP825685	39.70	BA.2.43	Omicron	29,771	0	99.3	366,263	2,817.3
213	SQ6301	2022-04	SRR29876891	PP825686	39.10	BA.2	Omicron	29,772	20.85	94.6	327,061	2,558.5
214	SQ6303	2022-04	SRR29876890	PP825687	41.20	BA.2	Omicron	29,734	28.76	93.8	238,759	1,743.1
215	SQ6307	2022-04	SRR29876889	PP825688	38.30	B.1.617.2	Delta	29,821	25	99.5	196,880	1,737.3
216	SQ6308	2022-04	SRR29876888	PP825689	43.30	BA.2.3	Omicron	29,576	46.49	91.9	154,097	789.96
217	SQ6310	2022-04	SRR29876887	PP825690	45.60	BA.2.3	Omicron	29,738	87.73	90.2	229,620	893.29
218	SQ6311	2022-04	SRR29876886	PP825691	40.80	BA.1.1	Omicron	29,819	7.47	97.8	324,671	2,308.9
219	SQ6316	2022-01	SRR29876885	PP825692	39.10	BA.2.38	Omicron	29,775	1.53	97.9	313,996	2,449.5
220	SQ6321	2022-05	SRR29876884	PP825693	44.40	BA.2	Omicron	29,426	65.25	90.3	132,006	498.47
221	SQ6324	2022-04	SRR29876882	PP825694	39.80	BA.2	Omicron	29,679	18.49	94.5	289,957	2,361.7
222	SQ6325	2022-04	SRR29876881	PP825695	38.80	BA.2	Omicron	29,729	5.85	96.4	210,095	1,540.1
223	SQ6330	2022-05	SRR29876880	PP825696	40	BA.2.12.1	Omicron	29,665	8.82	95.7	207,454	1,653.4
224	SQ6339	2022-05	SRR29876879	PP825697	38.40	BA.2	Omicron	29,789	12.72	96.5	361,239	3,407.8
225	SQ6360	2022-05	SRR29876878	PP825698	39.20	BA.2	Omicron	29,441	41.44	91.8	189,082	1,360.9
226	SQ6364	2022-05	SRR29876877	PP825699	39.70	BA.2.9	Omicron	29,441	52.91	91.1	208,067	1,797.8
227	SQ6366	2022-05	SRR29876876	PP825700	39.30	BA.2	Omicron	29,435	84.23	89.4	170,110	1,201.8
228	SQ6400	2022-02	SRR29876875	PP825701	43	BA.2.38	Omicron	29,657	17.08	94.6	566,559	3,000.6
229	ssq5500	2022-01	SRR29876874	PP825702	38.30	BA.1.17.2	Omicron	29,804	44.2	92.8	244,823	1,697.3

**Fig 1:**
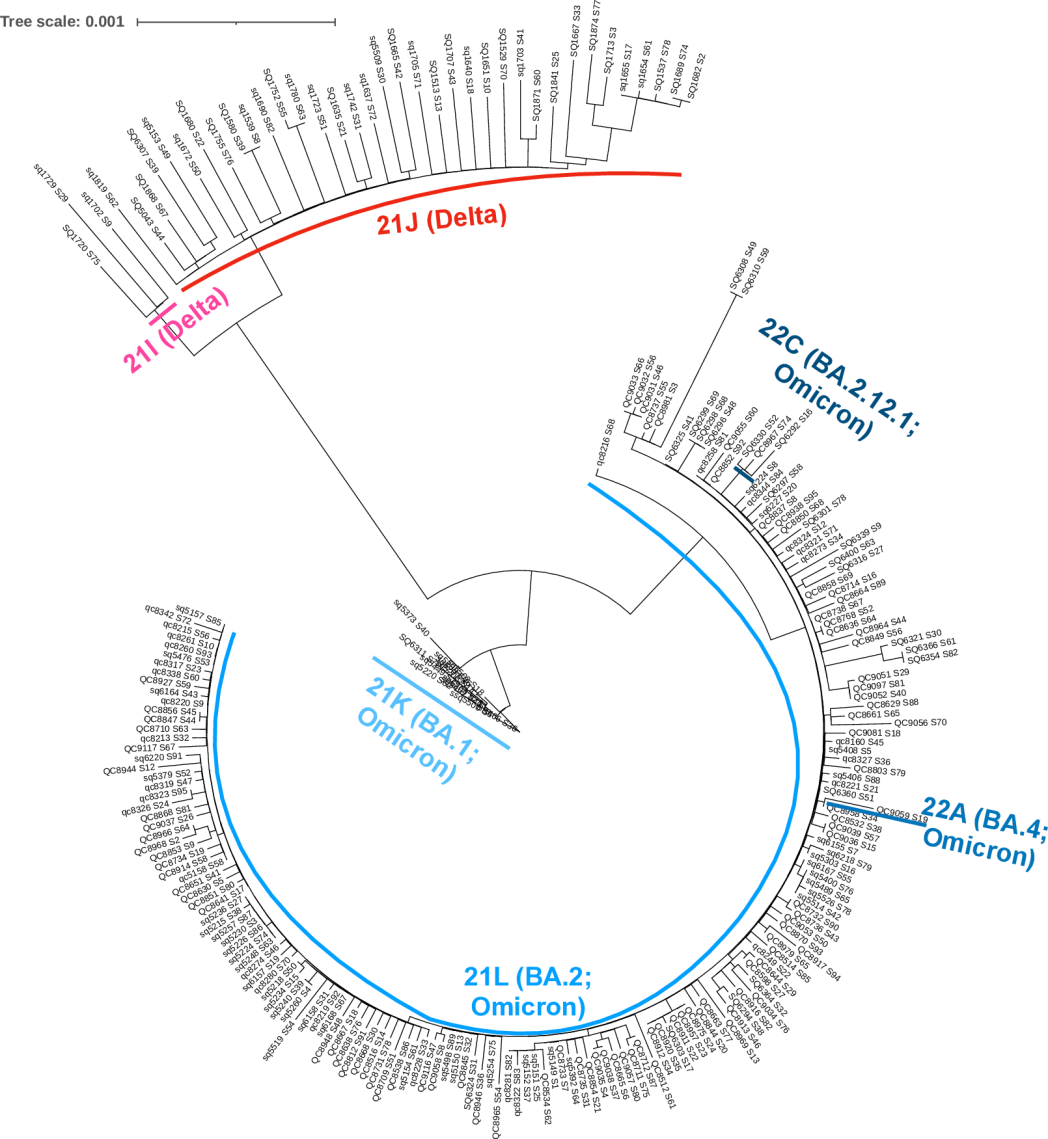
Phylogenetic analysis of complete SARS-CoV-2 genome sequences using GTR substitution model. The phylogenetic analysis showed a distinct clustering pattern among the circulating variants where the tree shows distinct clustering of Delta (red) and Omicron (blue).

Among the samples, 82.97% were Omicron with BA.2 and its sub-lineages (94.21%, *n* = 179/190) and 17.03% were Delta with B.1.617.2 (92.3%, *n* = 36/39). The phylogenetic tree showed the SARS-CoV-2 genomic diversity in Nepal (Fig. 1). This study provides insights and contributes valuable data for genomic surveillance in understanding and monitoring SARS-CoV-2 variants in Nepal.

## Data Availability

The GenBank accession numbers of the 229 sequences from this study are provided in Table 1. The raw reads are available in the SRA database under the BioProject number PRJNA1137145, and the accession IDs for these raw reads are also available in Table 1.
